# The Application of a Desktop NMR Spectrometer in Drug Analysis

**DOI:** 10.1155/2018/3104569

**Published:** 2018-09-19

**Authors:** Yonghong Zhong, Kejian Huang, Qiulian Luo, Suzhi Yao, Xiaofeng Liu, Ning Yang, Cuiwu Lin, Xuan Luo

**Affiliations:** ^1^School of Chemistry and Chemical Engineering, Guangxi University, Nanning 530004, China; ^2^Institute of Forensic Science, Public Security Department of Guangxi, Nanning 500012, China

## Abstract

A desktop NMR spectrometer was used to qualitatively analyze samples in drug-related cases in order to enhance the accuracy of the results and identify new drugs. Twelve known drugs and their derivatives were used to establish the parameters, conditions, and procedures for the methods and validate the feasibility and reliability of the methods. First, 1-D and 2-D NMR data for these 12 drugs and their derivatives were obtained in detail using a 600-MHz NMR spectrometer to create a data library. Next, some of these 12 drugs were analyzed using a Picospin 80 MHz desktop NMR spectrometer to set up the analytical procedure and method. With the procedure and method established, real case samples were analyzed and the data were compared to those obtained by a standard method. The results indicate that the desktop NMR spectrometer is a reliable and promising approach that can be used in criminology to quickly identify whether or not samples contain illegal drugs.

## 1. Introduction

Traditional detection methods for illicit drugs in the laboratory mainly include immunoassay [1], gas chromatography (GC) [2], high-performance liquid chromatography (HPLC), gas chromatography-mass spectrometry (GC-MS) [3, 4], and liquid chromatography-mass spectrometry (LC-MS) [5]. GC and HPLC are separation techniques, so they have certain disadvantages. For example, standards are required before these techniques can be used to identify components. In addition, in order to choose the correct chromatography column and experimental conditions, chromatography analysis needs to know the precise object of analysis. The advantage of mass spectrometry is the determination of the molecular weight of the substance. With a data library of mass spectra, MS data can provide preliminary structural information about compounds. When chromatography (GC and HPLC) is combined with MS, GC-MS and LC-MS have complementary advantages and show both qualitative and powerful quantitative analytical capabilities. However, due to the disadvantages of chromatography, GC-MS and LC-MS have many limitations for the analysis of unknown samples and new types of drugs. Additionally, advance information about the sample and time-consuming pretreatment is required [6]. As new drugs constantly appear, the components of illegal drugs become more complicated. The increasing abuse of illicit drugs requires a convenient and reliable analytical detection method for monitoring and qualitative analysis [7].

Nuclear magnetic resonance (NMR) is a highly efficient method for structure elucidation in the fields of chemistry and biology [8–11], and NMR has been widely applied for the qualitative analysis of new organic compounds [12]. NMR has many advantages over traditional detection methods [13]. One of the most important advantages is that NMR can quickly and accurately provide a wealth of structural information about compounds without destroying the properties of the compound [14, 15]. Recently, both liquid and solid NMR instruments have been used to study illicit drugs [16–19].

**Figure 1:**
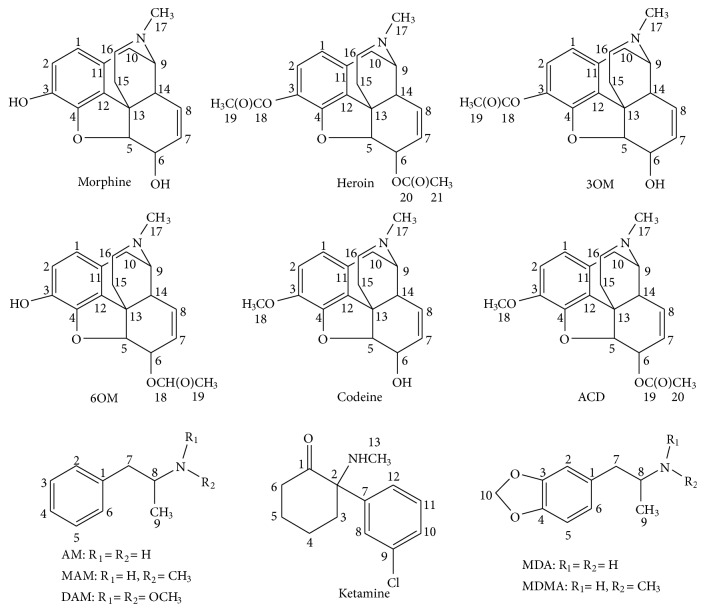
Chemical structures of twelve drugs.

In this paper, a set of feasibility and reliability methods will be established to analyze drug samples from criminology using a desktop NMR spectrometer. To achieve this goal, we used three well-known drug families (morphine, amphetamine, and ketamine), including morphine, heroin, 3-*O*-monoacetylmorphine (3OM), 6-*O*-monoacetylmorphine (6OM), codeine, acetylcodeine (ACD), amphetamine (AM), methamphetamine (MAM), 3,4-methylenedioxyamphetamine (MDA), 3,4-methylenedioxymethamphetamine (MDMA), *N*, *N*-dimethylamphetamine (DAM), and ketamine (KE) (Figure 1), to build an NMR database using a Bruker 600-MHz NMR spectrometer. Then we used a desktop NMR spectrometer to analyze the compounds and real drug samples from criminal cases. The results were compared to the data obtained by a standard method (GC-MS). Ultimately, we want to use the desktop NMR spectrometer to analyze drugs from case scenes.

## 2. Experimental

### 2.1. Chemicals and Reagents

Twelve standard drugs and illegal samples were authorized by the Public Security of Guangxi Province. Deuterium oxide (D_2_O), containing 0.05 wt.% 3-(trimethylsilyl) propionic-2,2,3,3-*d*_4_ acid sodium salt (TSP) as an internal standard, was purchased from the Aldrich Chemical Company (Missouri, USA). The coupling constants (*J*) are reported in Hertz (Hz). The experimental spectra of the standard drugs and illegal samples were measured using a Bruker AVANCE III HD600 NMR Spectrometer (Faellanden, Switzerland) with Topspin 3.2 software, which included a 5 mm liquid conventional probe and a 24 automatic sampler. The temperature of the probe was kept at 298 K for all experiments. The Picospin 80 MHz desktop NMR spectrometer and pipettes were purchased from Thermo Fisher Scientific (Madison, USA). The GC spectra were obtained using a QP2010 Ultra GC-MS (Shimadzu, Japan).

### 2.2. Experimental Parameters

The ^1^H NMR spectra were acquired using 64 K data points with a spectral width of 12019 Hz, the acquisition time of 2.73 s, the relaxation delay of 1 s, 16 scans, and a pulse width of 30°. The ^13^C NMR spectra were acquired using 64 K data points with a spectral width of 36232 Hz, the acquisition time of 0.91 s, the relaxation delay of 2 s, 1024 scans, and a pulse width of 30°. DEPT 90 and DEPT 135 spectra were performed using 64 K data points with a spectral width of 24038 Hz, the relaxation delay of 6.50 s, and 512 scans. The parameters used for the COSY spectra were 12019 Hz, an acquisition time of 0.21 s, a relaxation delay of 2 s, and 12 scans. HSQC experiments were recorded using the hsqcetgpsisp 2.2 pulse sequence with 24 scans. The spectral widths of the F1 (^13^C) dimension and the F2 (^1^H) dimension were 36232 Hz and 12019 Hz, respectively. The HMBC spectra were acquired using the hmbcgplpdqf pulse sequence with 80 scans. The spectral widths of the F1 (^13^C) dimension and the F2 (^1^H) dimension were 36232 Hz and 12019 Hz, respectively. Two-dimension selective HSQC (shsqcetgpsisp2.2) was performed in DAM with 24 scans.

The limits of detection for the 600-MHz ^1^H NMR spectra were acquired using 160 scans (8 min) and acquisition parameters similar to the abovementioned ^1^H NMR spectra. The ^1^H NMR spectra using the Picospin 80 desktop NMR spectrometer required 480 scans at 309 K with a bandwidth of 4 KHz.

The GC column was a DB-5ms capillary column (30 m × 0.2 mm × 0.25 *μ*m), and the carrier gas was ultrapure He (44 cm/sec). The GC temperature program started with an isothermal period at 80°C for 2 min followed by programming at 5°C/min to 200°C. The temperature was held at 200°C for 5 min, and then the temperature was increased at 30°C/min to 290°C, followed by a hold for 8 min. The temperatures of the injection port and mass selective detector interface were set at 280 and 250°C, respectively. The sample, 0.1 *μ*L, was injected in splitless mode initially, maintained for 1 min, and increased at a rate (split ratio = 20:1).

The mass spectrometer was operated in electron impact (EI) mode with a mass range of 40–500 U. The temperature of the MS source was 200°C. The electron ionization voltage was set at 70 eV.

### 2.3. Methods

The following masses were used for the drug standards: 10 mg for morphine, heroin, 3OM, 6OM, codeine, and DAM; 4 mg for amphetamine, MAM, KE, and MDMA; 8.6 mg for ACD; and 3.5 mg for MDA. These standard drugs were dissolved in 0.5 mL D_2_O, and the solutions were transferred into 5 mm NMR tubes for the NMR analysis. To achieve 2- or 5-fold dilution, respectively, 0.25- or 0.1-mL sample solutions were diluted with D_2_O to 0.5 mL.

For the desktop NMR analysis, the masses of morphine and MAM used were 50 mg. The masses of codeine, real crime sample 1 (S1), and real crime sample 2 (S2) were 80 mg. The samples for the desktop NMR experiments were dissolved in 0.5 mL of millipore water. The solution was filtered through a syringe filter (0.45 *μ*m) before injection into the desktop NMR spectrometer.

Case samples 1 (S1) and 2 (S2) weighed 5 mg and were dissolved in 5 mL methanol in a vial for the GC-MS analysis.

## 3. Results and Discussion

### 3.1. NMR Data Assignment

**Figure 2:**
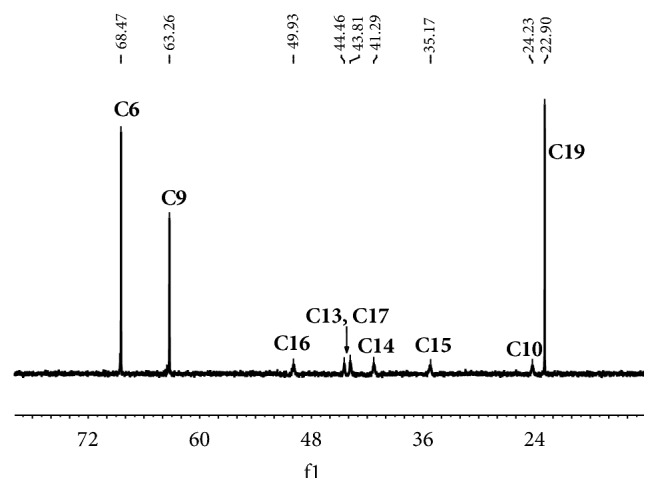
The ^13^C NMR spectrum of 3OM.

The^ 1^H NMR data for morphine and its derivatives are shown in Table 1. The integration values agree with the number of hydrogen atoms. The ^13^C NMR spectrum of morphine in D_2_O has six signals between 20 and 50. Comparison of the DEPT spectra shows that the signals at 23.81, 35.43, 41.34, 43.84, 44.38, and 50.10 can be assigned to C10, C15, C14, C17, C13, and C16, respectively. The structures of 3OM, 6OM, codeine, ACD, and heroin are similar to morphine. In the ^13^C NMR spectra of 3OM, 6OM, ACD, and heroin, the signals of C10, C13, C14, C15, C16, and C17 are also expected to be observed in the same region. However, these peaks are broad but very weak or even unobservable in an upfield shift. It is very likely that this finding is due to the influence of nitrogen atoms and the configuration of the structure [20]. In the ^13^C NMR spectrum of 3OM (Figure 2), the intensities of C10, C13, C14, C15, C16, and C17 were obviously weaker than the other peaks, which is quite different compared to morphine. Despite the weak intensity of the peaks in the carbon spectra of 3OM, 6OM, codeine, ACD, and heroin, it was still possible to draw conclusions about their structures using the 1-D and 2-D NMR spectra.

The ^1^H NMR chemical shifts for amphetamine, methamphetamine, DAM, MDA, and MDMA are summarized in Table 2. It is straightforward to assign the relationships between the carbons and protons based on the 1-D and 2-D spectra. In the selective HSQC spectrum of DAM, the two signals at 40.71 and 42.96 can be assigned to C11 and C12, which couple with the strong signal at 2.88 (H10 and H11).

These ^1^H NMR data for morphine and amphetamine derivatives have been reported in previous studies [21–23]. According to the research of Patrick A. Hays [24], different kinds of drugs have characteristic peaks in the ^1^H NMR spectra that do not overlap with those of other drugs. The signals of H19 from 3OM and 6OM appear at 2.36 and 2.20, respectively. The peaks at 2.36 and 2.17 can be assigned to H19 and H21, respectively, from heroin. H10 of MDA was observed at 5.98. The methyl signal from MDMA at 2.71 can be assigned to H11, and the signal at 5.99 can be assigned to H10. The results of this study show that each drug can be easily discriminated using a characteristic peak in the ^1^H NMR spectrum.

### 3.2. Limit of Detection Analysis

**Figure 3:**
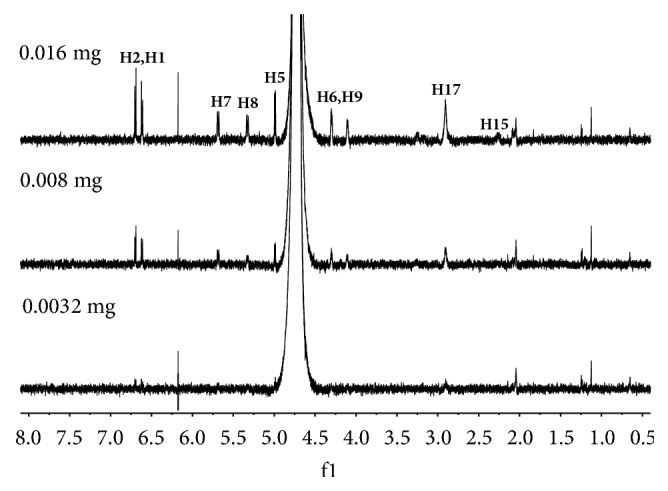
^1^H NMR spectra for different amounts of morphine.

**Figure 4:**
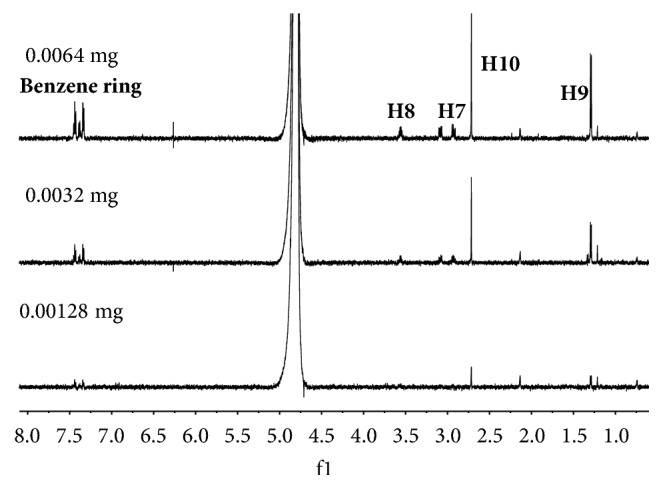
^1^H NMR spectra for different amounts of MAM.

To obtain the limits of detection for these 12 standard drugs using the 600-MHz NMR spectrometer, we used morphine, codeine, ACD, MAM, and DAM. The amounts of each drug in the NMR samples after each dilution are given in Table 3. The ^1^H NMR spectra of morphine over the last three dilutions are compared in Figure 3. The peaks in the spectrum of the sample containing 0.008 mg are still detectable. When the amount was decreased to 0.00128 mg, the signals of MAM (Figure 4) could not be discriminated from the experimental noise. For codeine, ACD, MAM, and MDA, the limits of detection were 0.008, 0.008, 0.0096, 0.0032, and 0.0028 mg (dissolved in 0.5 mL D_2_O in a 5-mm NMR tube), respectively. The results indicate that the limit of detection still differed from the limits of traditional methods (GC, LC, LC-MS, and GC-MS), but the NMR spectrometer can be qualitatively used to analyze new drugs at a very low concentration.

### 3.3. Desktop NMR Spectrometer Analysis

**Figure 5:**
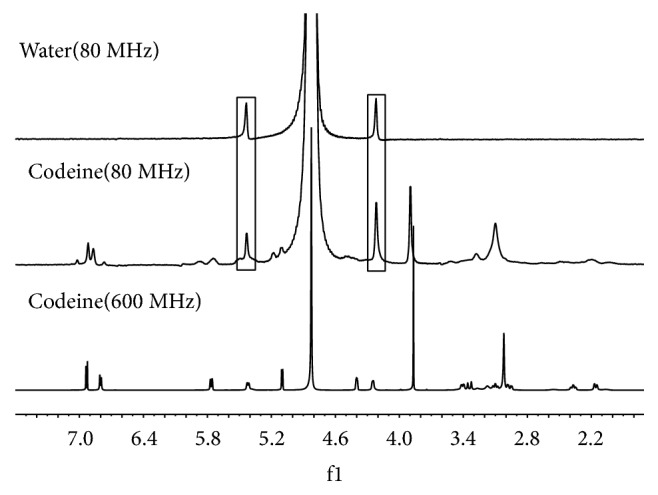
The 80 MHz ^1^H NMR spectra of water and codeine.

**Figure 6:**
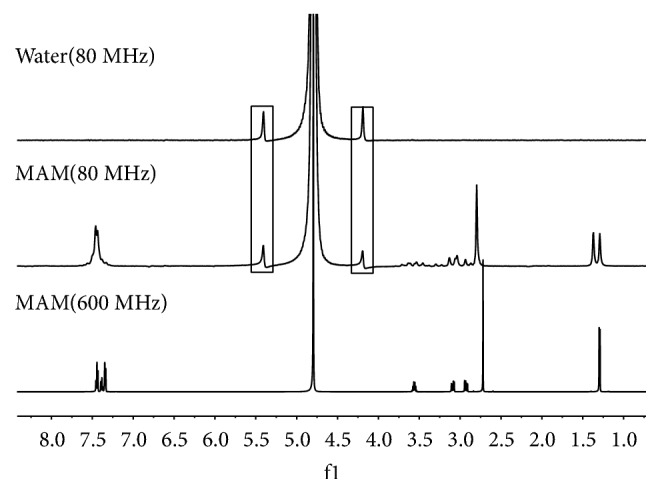
The 80 MHz ^1^H NMR spectra of water and MAM.

The limit of detection is one of the important performance parameters for an instrument. The specifications of the Picospin 80 MHz desktop NMR spectrometer show that the detection limit is 0.1 mol. Figures 5 and 6 show the desktop NMR spectra of the codeine and MAM standard samples. The two symmetrical peaks observed at 4.19 and 5.40 are background peaks from the desktop NMR spectrometer. The methyl and benzyl ring peaks of the codeine and MAM standard samples agreed with the data from the 600 MHz ^1^H NMR spectra.

**Figure 7:**
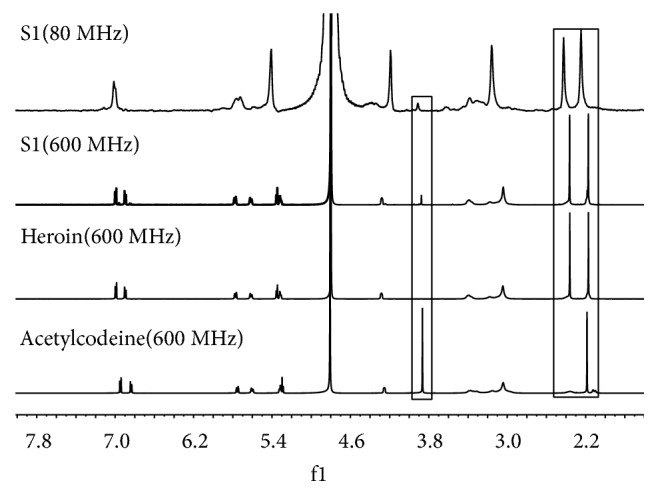
The ^1^H NMR spectra of S1, heroin, and acetylcodeine.

**Figure 8:**
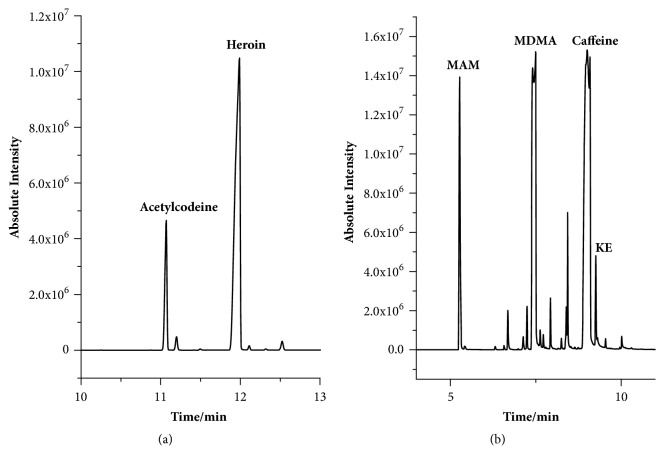
The GC chromatograms for S1 (a) and S2 (b).

S1 and S2 are real samples from crime scenes.  Figure 7 shows the results for S1. The characteristic peaks in the spectrum indicate that the components are morphine derivatives. The signals appearing at 2.25 and 2.42 for S1 are the methyl of heroin. Another characteristic signal appeared at 3.91 and was assigned to the H18 of acetylcodeine. The results agreed well with the GC chromatogram (Figure 8(a)), but the chemical shifts from the desktop NMR spectra showed slight differences. Although the concentration of acetylcodeine was lower than that of heroin, the methyl protons of acetylcodeine could still be observed in the ^1^H NMR spectrum.

**Figure 9:**
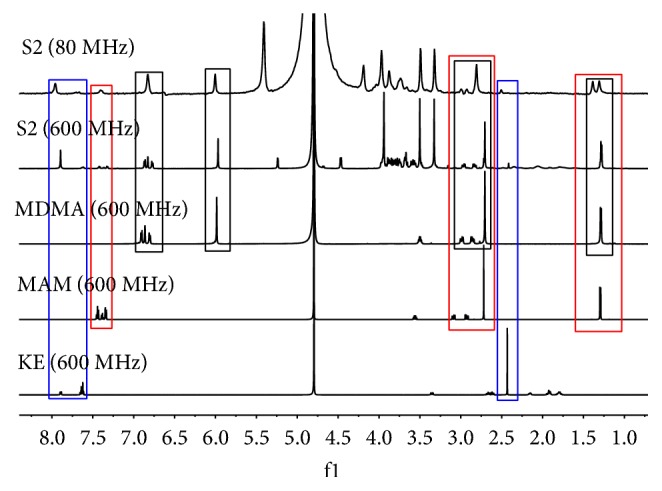
The ^1^H NMR spectra for S2, MDMA, MAM, and KE.

Based on the standard ^1^H NMR spectra, the characteristic signals at 1.34, 2.81, and 7.38 were assigned to MAM (Figure 9). H11 of MDMA appears at 6.00 due to the oxygen atom, and the chemical shift of the benzyl ring protons is 6.85. The characteristic signal of the methyl group of KE was observed at 2.43, while the benzyl ring signals were observed at 7.63 and 7.89. However, the signals of ketamine observed in the spectrum are very weak, indicating that the content of ketamine in S2 is low. Three strong peaks between 3 and 4 in the ^1^H NMR spectrum indicate that there may be three methyl groups. On the basis of the GC chromatogram (Figure 8(b)), S2 contains MAM and MDMA. The content of ketamine is lower than that of the other components. Meanwhile, the chemical structure of caffeine contains three methyl groups attached to three different nitrogen atoms.

These experiments suggest that the desktop NMR spectrometer can effectively detect drugs by observing the methyl and benzyl ring peaks of standard samples. In addition, the desktop NMR can also provide information about unknown substances.

## 4. Conclusion

In this work, we successfully obtained a library containing the spectra of 12 standard drugs using a 600 MHz NMR spectrometer and mastered the desktop NMR spectrometer. Both morphine and amphetamine derivatives had low limits of detection using the 600-MHz ^1^H NMR spectra. Two real case samples were analyzed to verify the reliability of the desktop NMR spectrometer. Based on the characteristic peaks in the ^1^H NMR spectra of the standard drugs, the results showed that one sample mainly contained morphine and acetylcodeine, while the other contained MAM and MDMA. In conclusion, the desktop NMR spectrometer is an effective qualitative method for the analysis of drugs. We hope that desktop NMR spectrometers can be applied in case scenes in the future to analyze drugs from crimes.

## Figures and Tables

**Table 1:** ^1^H NMR data for morphine, heroin, 3OM, 6OM, codeine, and ACD in 0.5 mL D_2_O.

**Atom**	**Morphine**	**Heroin**	**3OM**	**6OM**	**Codeine**	**ACD**
H1	6.71, d	6.90, d	6.85, d	6.74, d	6.80, d	6.84, d
H2	6.79, d	6.99, d	6.97, d	6.82, d	6.93	6.94, d
H5	5.09, d	5.31, d	5.14, d	5.24, d	5.10, d	5.30, d
H6	4.20, d	5.34, d	4.42, d	5.30, m	4.40, d	5.32, d
H7	5.76, d	5.62, d	5.78, d	5.58, d	5.76, d	5.75, d
H8	5.41, d	5.77, d	5.44, d	5.77, d	5.42, d	5.60, d
H9	4.24, s	4.28, s	4.26, s	4.20, s	4.25, s	4.25, s
H10	2.95,3.31, d	3.04,3.39, s	3.04,3.36, m	2.98,3.25, d	3.40,2.96, d	3.04,3.32, s
H14	3.02, s	3.18, s	3.04, m	3.14, s	3.02, s	3.15, s
H15	2.36,2.15, d	2.36,2.17, s	2.21,2.39, m	2.13,2.36, m	2.16, 2.37, d	2.10,2.36, d
H16	3.40,3.10, d	3.04,3.39, s	3.17,3.35, m	3.38,3.08, m	3.10,3.41, s	3.37,3.04, m
H17	3.02, s	3.04, s	3.04, s	3.04, s	3.02, s	3.04, s
H18	N/A	N/A	N/A	N/A	3.87, s	3.87, s
H19	N/A	2.17, s	2.36, s	2.20, s	N/A	N/A
H20	N/A	N/A	N/A	N/A	N/A	2.19, s
H21	N/A	2.36, s	N/A	N/A	N/A	N/A

**Table 2:** ^1^H NMR chemical shifts for AM, MAM, KE, DAM, MAD, and MDMA.

**Atom**	**AM**	**MAM**	**DAM**	**MDA**	**MDMA**	**KE**
H2	7.33, d	7.34, d	7.37, d	6.86, s	6.86, s	N/A
H3	7.44, t	7.44, t	7.45, t	N/A	N/A	1.92,3.35, m
H4	7.38, t	7.38, t	7.40, t	N/A	N/A	1.80,1.92, m
H5	7.44, t	7.44, t	7.45, t	6.90, d	6.90, d	1.80,2.15, m
H6	7.33, d	7.34, d	7.37, d	6.80, d	6.80, d	2.65, d
H7	2.96, m	2.92,3.10, m	2.91,3.16, m	2.87, m	2.86,2.98, m	N/A
H8	3.65, m	3.56, m	3.73, m	3.60, m	3.50, m	7.90
H9	1.32, d	1.30, d	1.27, d	1.32, d	1.28, d	N/A
H10	N/A	2.72, s	2.88, s	5.98, s	2.71, s	7.63, m
H11	N/A	N/A	2.88, s	N/A	5.99, s	7.63, m
H12	N/A	N/A	N/A	N/A	N/A	7.63, m
H13	N/A	N/A	N/A	N/A	N/A	2.43, s

**Table 3:** The amounts (mg) of morphine, codeine, ACD, MAM, and MAD used in the dilution experiment.

**Sample**	**First**	**Second**	**Third**	**Fourth**	**Fifth**	**Sixth**	**Seven**
Morphine	10	2	0.4	0.08	0.016	0.008	0.0032
Codeine	10	2	0.4	0.08	0.016	0.008	0.0032
ACD	1.2	0.24	0.048	0.0096	0.0048	0.00192	N/A
MAM	4	0.8	0.16	0.032	0.0064	0.0032	0.00128
MDA	3.5	0.7	0.14	0.028	0.0056	0.0028	0.00112

## Data Availability

The data used to support the findings of this study are available from the corresponding author upon request.

## Supplementary Materials

Supplementary Materials The supplementary materials include Picospin 80 desktop NMR spectrometers, the 600 MHz NMR spectra of five samples (including morphine, heroin, 6-*O*-monoacetylmorphine, acetylcodeine, and* N,N*-dimethylamphetamine), and the dilution experiment spectra for acetylcodeine and 3,4-methylenedioxyamphetamine.
