# Supplementary material for: The Application of a Desktop NMR Spectrometer in Drug Analysis
Source: Int J Anal Chem. 2018 Sep 19;2018:3104569. doi: 10.1155/2018/3104569 (PMC6169242; doi:10.1155/2018/3104569)

**Supplementary Material**

1. The ^1^H NMR acquisition parameters for Picospin 80 desktop NMR spectrometer.

tx frequency: 82.347507 MHz

auto tx offset: 0 Hz

scans: 480

pulse length: 30 μs

acquisition points: 3000

recovery delay: 500 μs

recycle delay: 8 s

bandwidth: 4 kHz

post filter attenuatic: 11

zero filling: 8192

phase correction: 0 degrees

exp. apodization: 0 Hz

max time to plot: 250 ms

min freq. to plot: -2000 Hz

max freq. to plot: 2000 Hz

max plot points: 400

2. The ^13^C NMR spectrum for morphine (150 MHz, D_2_O).


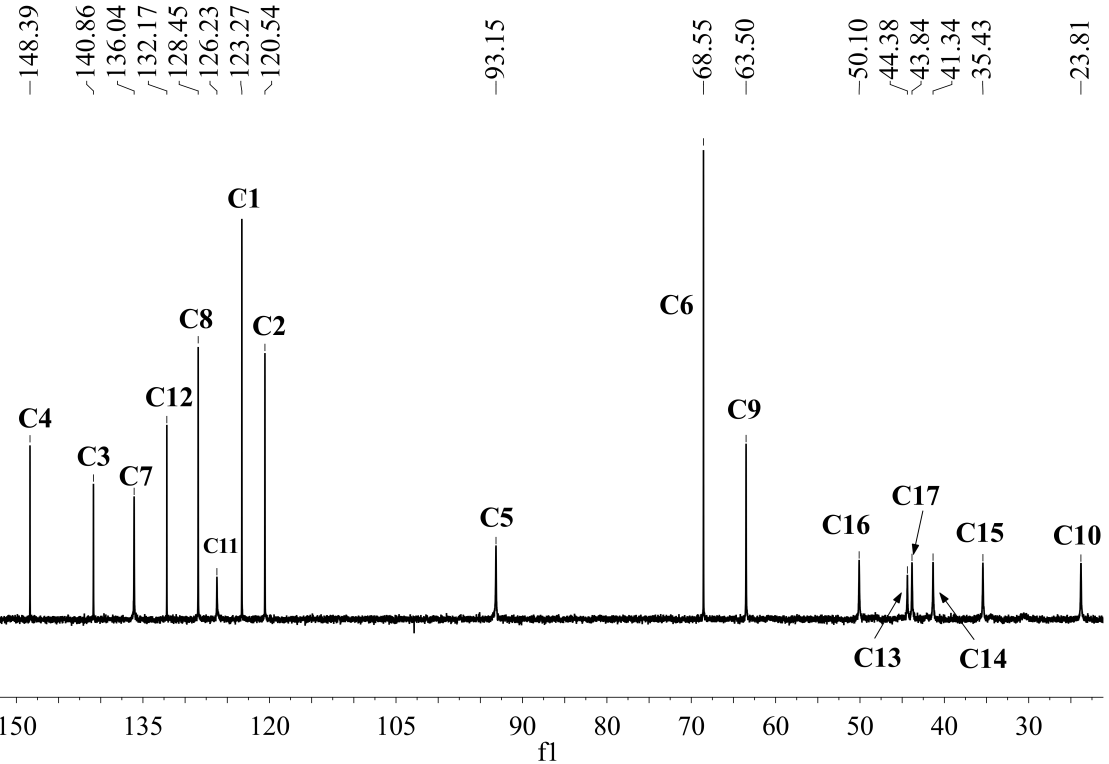


3. The ^13^C NMR spectrum for heroin (150 MHz, D_2_O). The intensities of C10, C13, C14, C15, C16, and C17 were obviously weaker than the other peaks, which is quite different compared to morphine.


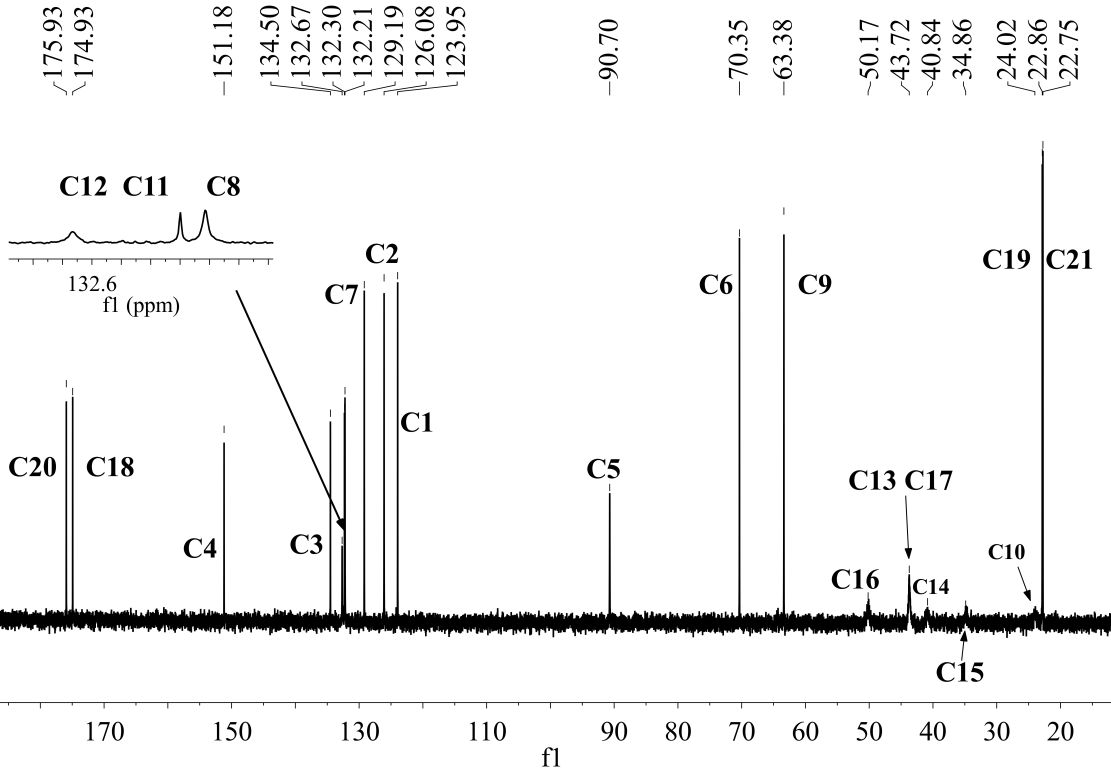


4. The ^13^C NMR spectrum for 6-*O*-monoacetylmorphine (6OM, 150 MHz, D_2_O). The intensities of C10, C14 and C15 were obviously weaker than the other peaks, which is quite different compared to morphine.


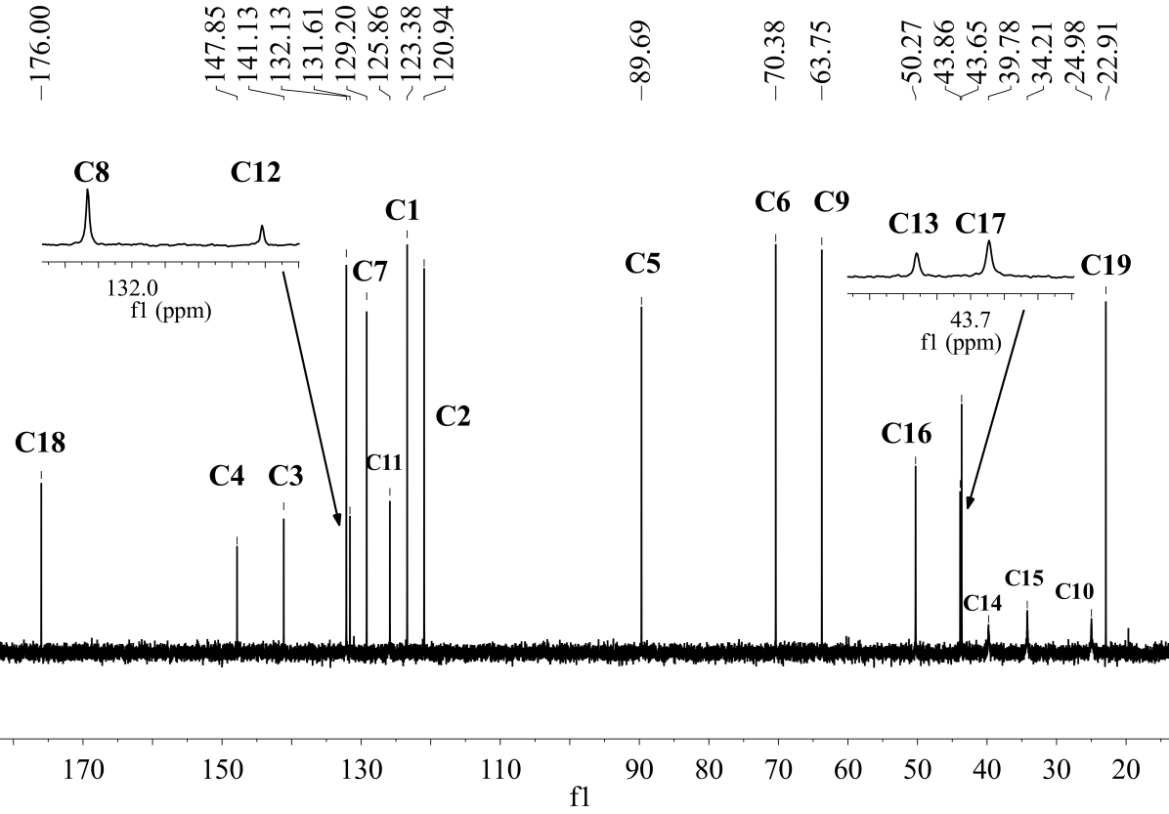


5. The ^13^C NMR spectrum for acetylcodeine (ACD, 150 MHz, D_2_O). The intensities of C10, C13, C14, C15, C16, and C17 were obviously weaker than the other peaks, which is quite different compared to morphine.


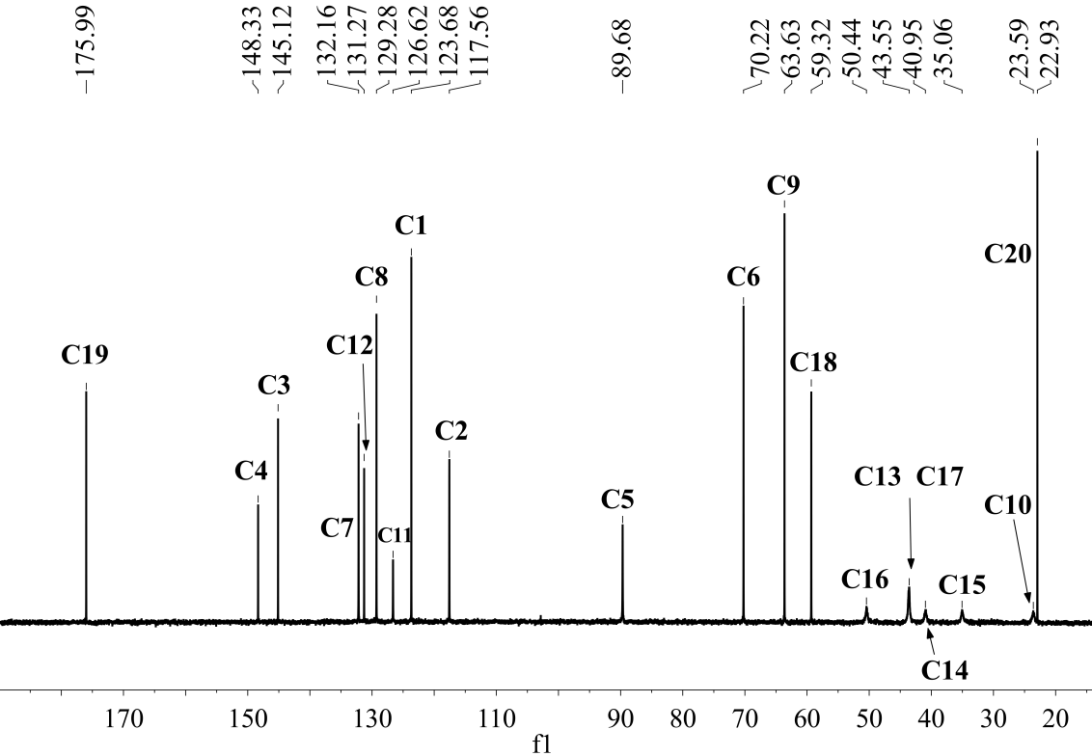


6. Spectra information for *N,N*-dimethylamphetamine (DAM).

6.1 The ^1^H NMR spectrum of DAM (600 MHz, D_2_O).


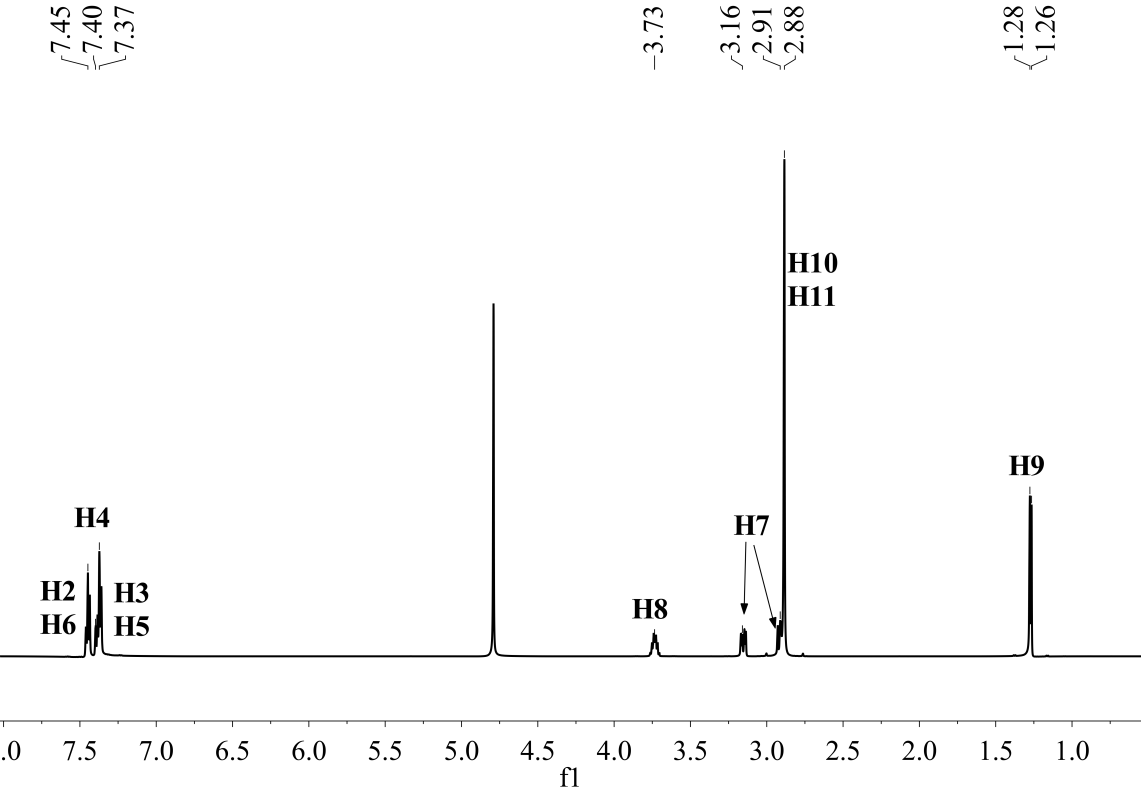


6.2 The ^13^C NMR spectrum of DAM (150 MHz, D_2_O). The intensities of 40.71 and 42.96 were obviously weaker than the other peaks.


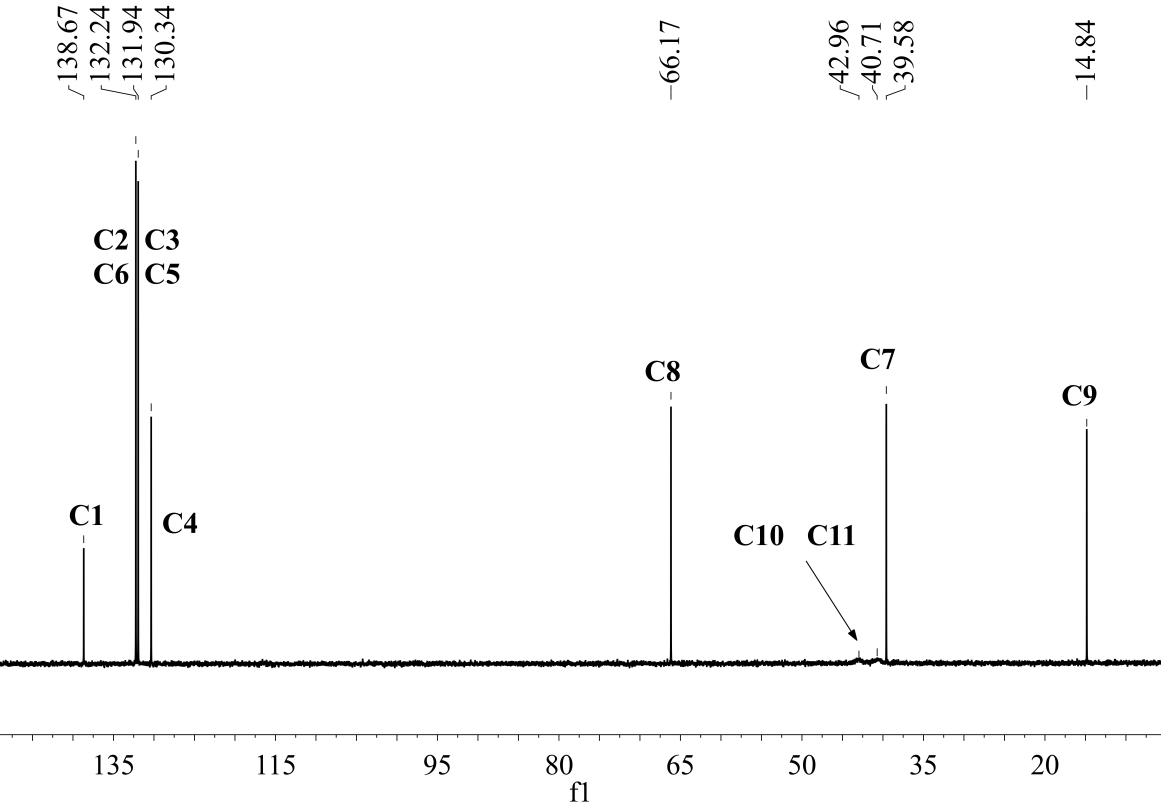


6.3 In the selective HSQC spectrum of DAM(600 MHz, D_2_O), the two signals at 40.71 and 42.96 can be assigned to C11 and C12, which couple with the strong signal at 2.88 (H10 and H11).


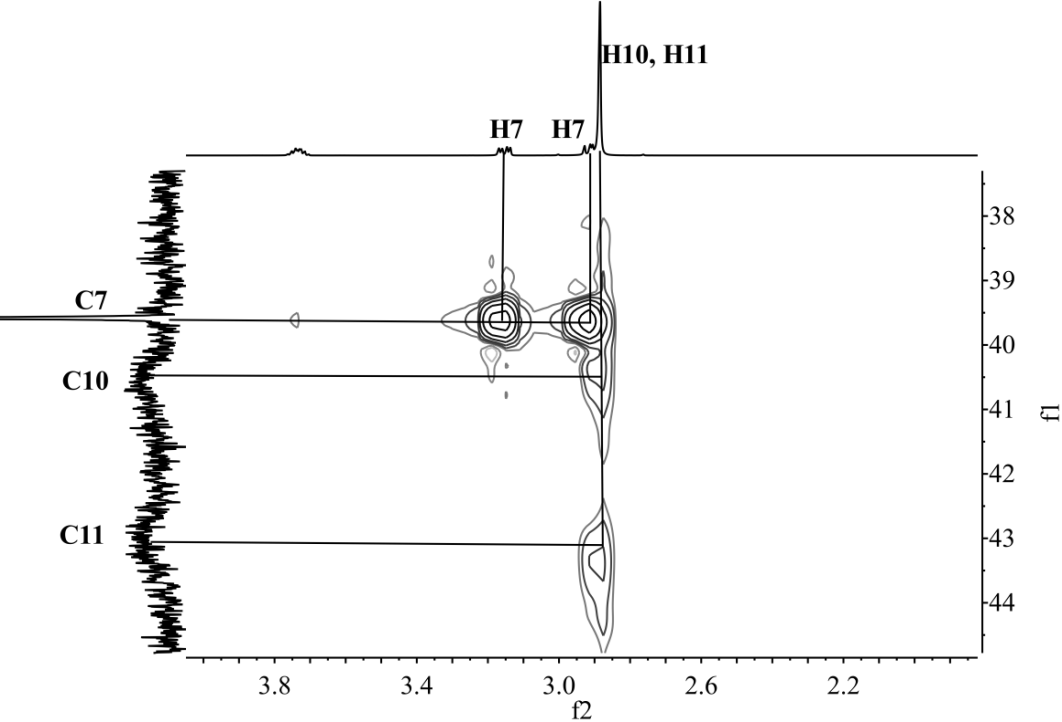


7. Dilution experiments spectra for detection limit of acetylcodeine (ACD, 600 MHz, D_2_O). The peaks in the spectrum of the sample containing 0.0096 mg are still detectable. When the amount was decreased to 0.0048 mg, the signals of ACD could not be discriminated from the experimental noise. The results indicate that the limits of detection were 0.0096 mg (dissolved in 0.5 mL D_2_O in a 5-mm NMR tube).


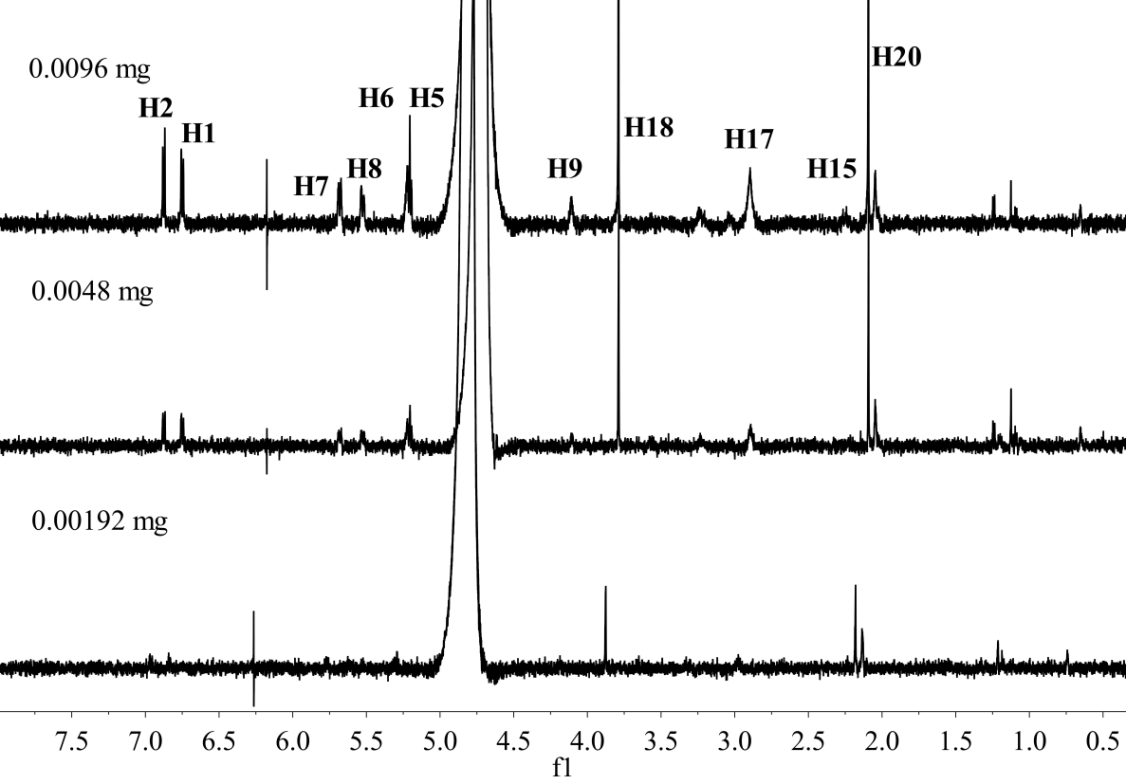


8. Dilution experiments spectra for detection limit of 3,4-methylenedioxyamphetamine (MDA, 600 MHz, D_2_O). The peaks in the spectrum of the sample containing 0.0028 mg are still detectable. When the amount was decreased to 0.00112 mg, the signals of MDA could not be discriminated from the experimental noise. The results indicate that the limits of detection were 0.0028 mg (dissolved in 0.5 mL D_2_O in a 5-mm NMR tube).


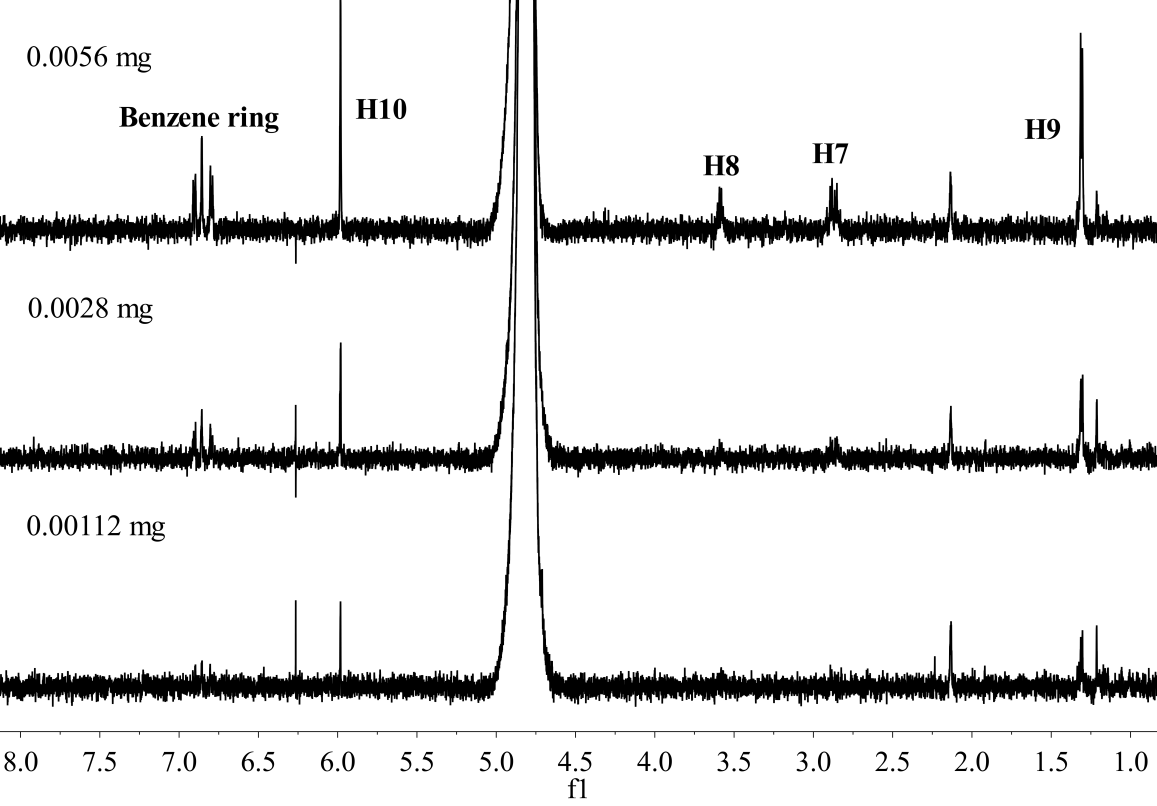

Supplement: Supplementary Materials — The supplementary materials include Picospin 80 desktop NMR spectrometers, the 600 MHz NMR spectra of five samples (including morphine, heroin, 6-O-monoacetylmorphine, acetylcodeine, and N,N-dimethylamphetamine), and the dilution experiment spectra for acetylcodeine and 3,4-methylenedioxyamphetamine. [file 3104569.f1.docx]
